# Comparison of differences in immune cells and immune microenvironment among different kinds of oncolytic virus treatments

**DOI:** 10.3389/fimmu.2024.1494887

**Published:** 2024-11-11

**Authors:** Xiaoke Wu, Shaokuan Fang

**Affiliations:** Department of Neurology, Neuroscience Centre, The First Hospital of Jilin University, Changchun, China

**Keywords:** oncolytic virus, tumor, treatment, immune cell, tumor microenvironment

## Abstract

Oncolytic viruses are either naturally occurring or genetically engineered viruses that can activate immune cells and selectively replicate in and destroy cancer cells without damaging healthy tissues. Oncolytic virus therapy (OVT) represents an emerging treatment approach for cancer. In this review, we outline the properties of oncolytic viruses and then offer an overview of the immune cells and tumor microenvironment (TME) across various OVTs. A thorough understanding of the immunological mechanisms involved in OVTs could lead to the identification of novel and more effective therapeutic targets for cancer treatment.

## 1 Introduction

Treatment options for tumors have expanded significantly in recent years, driven by an enhanced understanding of the immunologic mechanisms underlying these diseases (1, 2). These options now include traditional surgical treatments and chemoradiotherapy, as well as innovative approaches like immune checkpoint inhibitors, chimeric antigen receptor T-cells, adoptive cell immunotherapy, monoclonal antibodies, nanoantibodies, and other forms of immunotherapy (3–7). While surgical resection remains the most effective treatment for cancer and can substantially relieve symptoms, it is often not viable for patients in advanced stages of the disease. Moreover, surgery often leads to distant metastasis and local recurrence post-operatively (8). Chemoradiotherapy can decelerate cancer growth and prolong survival, however, it poses severe risks due to its damaging effects on normal cells while targeting cancer cells (9, 10). Immunotherapies, despite their potential, benefit only a select group of patients due to various immunosuppressive factors such as immune system suppression, deficiencies in cytokine types, reduced activity of tumor-infiltrating lymphocytes, impaired function of antigen-presenting cells, and weakened effector T-cell activity (11–13). Oncolytic viruses have emerged as one of the most promising treatments to address these challenges (14). These viruses are capable of selectively replicating within tumor cells, delivering multiple eukaryotic transgene payloads, inducing immunogenic cell death, enhancing antitumor immunity, and exhibiting a safety profile that generally does not overlap with other cancer treatments (15, 16). Recent clinical trials of oncolytic virus therapy (OVT) have shown minimal severe adverse reactions, with only local injection site reactions and low-grade systemic symptoms noted (17–20). In 2005, Chinese regulators approved the first genetically modified oncolytic adenovirus for the treatment of nasopharyngeal carcinoma in combination with chemotherapy, marking the world’s first oncolytic virus approved for clinical cancer treatment (21).

**Figure 1:**
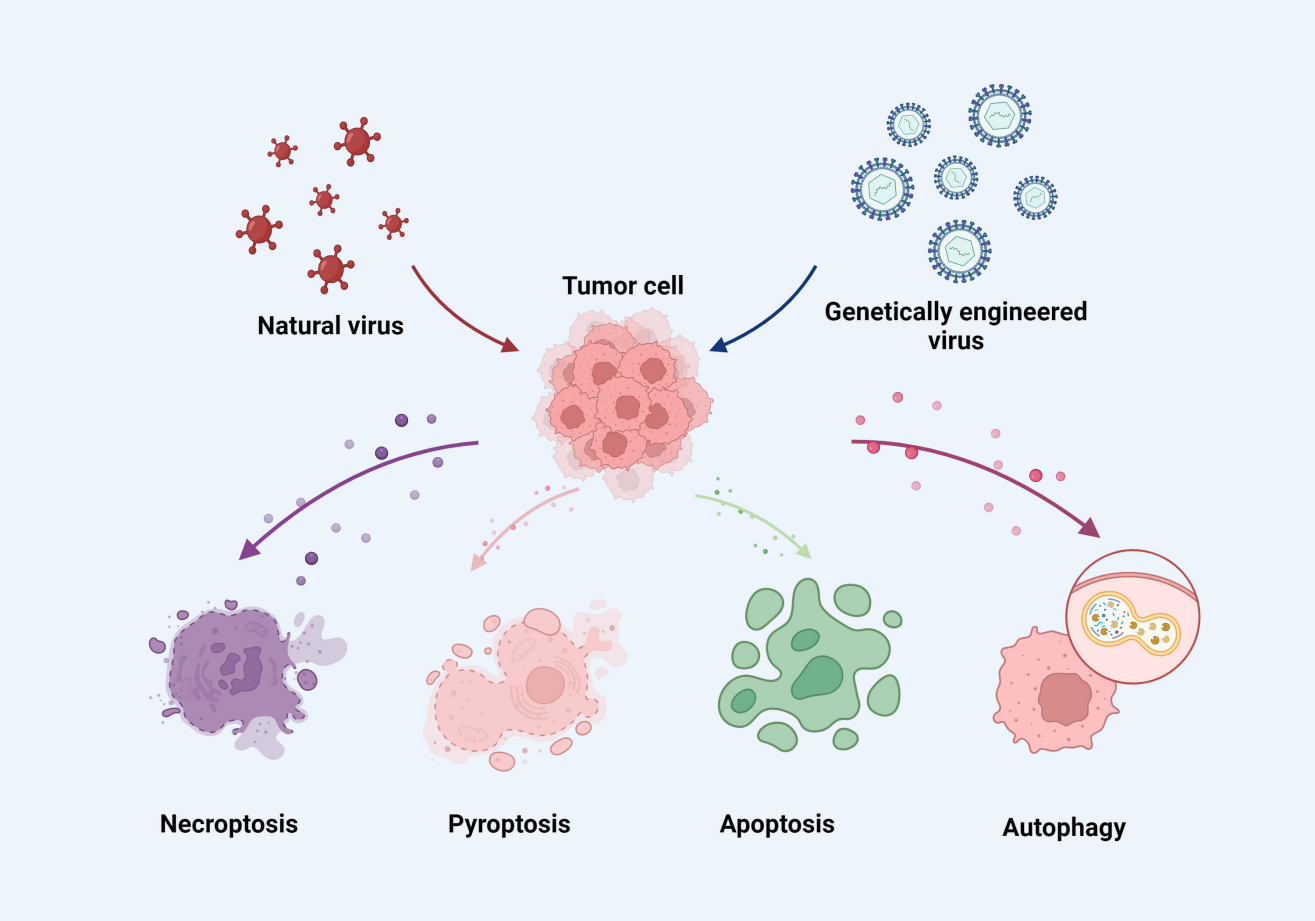
Oncolytic viruses induce necroptosis, pyroptosis, apoptosis and autophagy in cancer cells.

Oncolytic viruses, either genetically modified or naturally occurring, have the capability to activate immune cells that specifically target and destroy cancer cells while sparing healthy cells (22). These viruses selectively infect tumor cells harboring mutations associated with malignancies (23), and often have modifications in or deletions of certain viral genes essential for replication in normal cells but not in tumor cells (24). This selective infection is facilitated by the viruses’ ability to bind specifically to certain receptors on tumor cells, enhanced by altering the virus’s tropism (25). The viral genes are controlled by tumor-specific and/or tissue-specific gene promoters, enhancing the safety by confining viral replication to tumor cells (26, 27). Tumor cells frequently exhibit dysfunctional immune response signaling pathways, which support the virus’s replication and proliferation within them (28, 29). Oncolytic viruses inhibit the production of host cellular products while enhancing viral product synthesis within the tumor cells. When oncolytic viruses lyse tumor cells, they release large quantities of tumor antigens that are then processed by antigen-presenting cells, triggering T-cells to target both infected and uninfected tumor cells (30, 31). Upon infecting tumor cells, the viruses can either directly affect immune cells or stimulate the tumor cells to produce more cytokines, thereby enhancing the immune system to eliminate any remaining tumor cells by phagocytosing them (32). Oncolytic viruses induce various cell death pathways in cancer cells, including necroptosis, pyroptosis, apoptosis, and autophagy (**Figure 1**) (33–36). For example, the N-terminal gasdermin domain (GSDMNT), when delivered into tumor cells via a recombinant adeno-associated virus, induces pyroptosis, offering significant therapeutic potential (34). Similarly, the oncolytic vaccinia virus, when armed with the aphrocallistes vastus lectin gene, can alter the metabolism of hepatocellular carcinoma cells, increase reactive oxygen species (ROS) production, and promote apoptosis, all contributing to its antitumor effects (37). OVT works by killing cancer cells using viruses that have a selective replication function (1, 38). There are variations among oncolytic viruses in terms of how they interact with immune cells and the tumor microenvironment (TME) during treatment (39, 40). In this paper, we provide a detailed overview of the immune cells, TME, and oncolytic viruses, and discuss the variations in TME and immune responses observed with current OVTs.

## 2 Origins and characteristics of oncolytic viruses

For nearly a century, researchers have been studying viruses. The first viruses identified were the foot-and-mouth disease virus from animals and the contagium vivium fluidum from plants, both discovered in 1898 (41, 42). Additionally, the yellow fever virus was first identified in 1901 and associated with human disease (43, 44). Advances in technology and an enhanced understanding of virus morphology and virulence have enabled ex vivo virus replication. This has facilitated the linking of several diseases, including rabies and influenza, to specific viruses (45, 46). Subsequent research has provided a detailed understanding of viral species, including their structure and biological characteristics (47). Simultaneously, researchers discovered that the virus may be utilized to cure tumors in addition to causing infectious diseases (48, 49).

Studies have documented cases of tumor regression following viral infection (49, 50). However, these remissions were typically short-lived, generally lasting only one to two months. In 1922, Levaditi and colleagues observed that the vaccinia virus could inhibit various cancers in mice and rats (51). In 1950, Pack reported remissions in patients with metastatic melanoma who were treated with the rabies virus. Additionally, patients with hematological malignancies such as leukemia or lymphoma experienced remission following infections with chickenpox or influenza (52, 53). Concurrent regressions of leukemia, Hodgkin’s disease, and Burkitt’s lymphoma have also been noted during measles infections, suggesting that under the right circumstances, certain viruses can target malignancies effectively without endangering the patient (54, 55). For instance, in a study of juvenile diffuse intrinsic pontine glioma treated with the oncolytic DNX-2401 virus, magnetic resonance imaging showed a reduction in tumor volume in 75% of the cases, and 66.7% of the patients achieved stable disease (56). Furthermore, a study involving 19 patients with recurrent glioblastoma treated with the oncolytic virus DNX-2401 demonstrated its safety and feasibility; notably, one patient achieved complete regression and was still alive eight years later (57).

**Figure 2:**
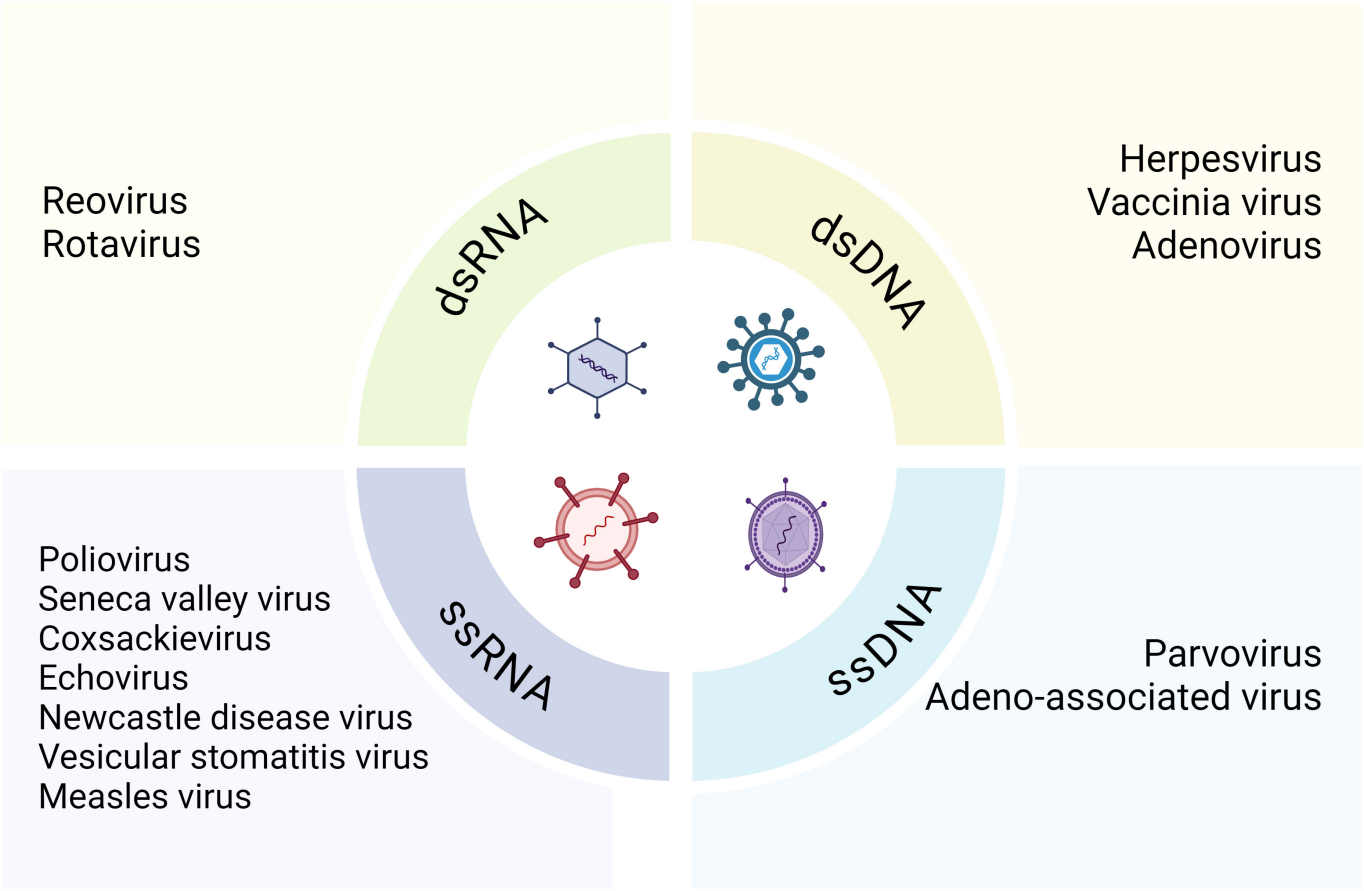
Classification of the oncolytic viruses.

Various viruses have been evaluated for their oncolytic properties in human tumor cell lines prior to their progressive utilization in clinical trials and other therapeutic contexts (58). In models of KRAS-mutated colorectal cancer, such as HCT116 colon cancer cells and patient-derived peripheral blood mononuclear cells, the oncolytic reovirus (pelareorep) exploits host autophagic machinery to enhance its proliferation and achieve selective oncolysis (59). In a phase 1 dose-escalation study, patients with newly diagnosed high-grade glioma who were treated with neural stem cell-delivered engineered oncolytic adenovirus showed promising safety and efficacy (60). Originally, hepatitis viruses were used to treat hematological malignancies and leukemia symptoms remitted in patients infected with adenovirus or EB virus (61). Subsequently, Alice Moore solidified the existence of an oncolytic virus in 1950 through *in vivo* tumor models and clinical and preclinical research (62). Oncolytic viruses, defined as naturally occurring or genetically modified viruses, selectively replicate in tumor cells, destroying them without harming healthy tissues and can also stimulate systemic or localized anti-tumor immunity (14, 63). These viruses target specific cell surface receptors overly expressed by cancer cells and naturally prefer cell surface proteins, enabling them to bind these receptors and penetrate the cells (64, 65). Oncolytic viruses are divided into two main categories: RNA and DNA viruses, including single-stranded (ssRNA and ssDNA) and double-stranded (dsRNA and dsDNA) viruses (**Figure 2**) (66).The most common RNA viruses used are measles and coxsackie virus group B, while commonly used DNA viruses include adenovirus, herpes simplex virus 1, and vaccinia virus. Except for vaccinia, DNA viruses have longer replication cycles than RNA viruses and replicate in the nuclei of infected cells (67, 68). Oncolytic viruses can also be classified into genetically engineered and naturally occurring strains. Engineered strains exhibit decreased pathogenicity, enhanced tumor expression, and increased lethality compared to wild-type strains (69). Mutations in the presence or lack of certain viral genes that are necessary for the virus to multiply in healthy cells but not in tumor cells (24). Genetically engineered oncolytic viruses are also designed to improve targeting selectivity for tumor cells and host cells with cancer-related mutations (70). Oncolytic viruses are intended to identify tumor-upregulated receptors, allowing the virus for an improved fidelity (71).To increase safety and limit viral replication to tumor cells, their principal genes are cloned into tumor-specific or tissue-specific promoters (26, 27). Besides their direct oncolytic activity of preferentially replicating within and destroying cancer cells, oncolytic viruses are highly effective in triggering immune responses against both the tumor cells and themselves. They are capable of inducing both local and systemic anti-tumor immunity, utilizing immune evasion strategies employed by cancer cells (72).

## 3 Tumor-associated immune cells and immune microenvironment

### 3.1 Tumor-associated immune microenvironment

Since the 1970s, there has been accumulating evidence that TME plays a crucial role in tumor development, either by facilitating or hindering it (73, 74). Additionally, Stephen Paget’s “seed and soil theory,” introduced in 1989, conceptualized the interplay between cancer cells and the TME (75, 76).There is increasing evidence that tumor progression requires the recruitment and reprogramming of adjacent normal cells. The TME consists of a complex network of exosomes, the extracellular matrix (ECM), and stromal cells. During the occurrence and progression of cancer, tumor cells interact with surrounding cells to influence the cancer’s spread, proliferation, immune evasion, and chemoresistance within the TME; concurrently, these components undergo dynamic changes (77, 78). Different tumor locations and types exhibit specific TMEs, notable for their heterogeneity and dynamic changes. In colorectal cancer, stratifying patients based on the interindividual variability of the TME revealed differences in the immune evasion tactics employed by cancer cells among various TME subtypes (79). A cell-level analysis in prostate cancer samples studied the cell states associated with tumorigenesis, focusing on epithelial cell subsets, stromal cells, and the TME. This analysis found that ERG- cells show heterogeneity with luminal epithelial cells and differ from ERG+ tumor cells, potentially inducing a characteristic TME response (80).

**Figure 3:**
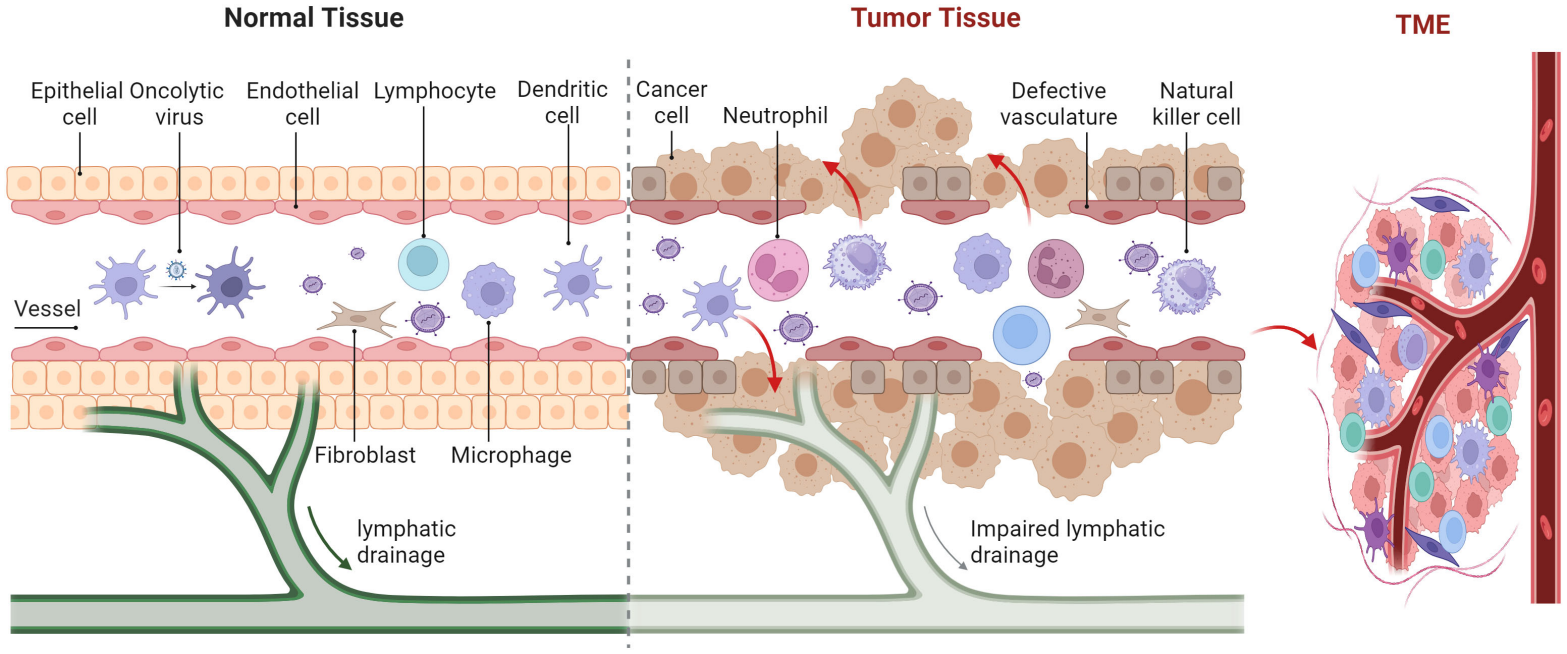
Various cells and oncolytic viruses in the tumor microenvironment.

The TME is comprised of stromal cells including cancer-associated fibroblasts (CAFs), macrophages, dendritic cells (DCs), natural killer (NK) cells, neutrophils, lymphocytes, endothelial cells, as well as chemokines, matrix metalloproteinases (MMPs), integrins, and other secreted molecules (**Figure 3**) (81). The TME contains various signaling molecules and pathways that contribute to immune suppression and angiogenic responses (82–84). The methyltransferase-like 3 associated with RNA N6-methyladenosine (m6A) modification is strongly activated by lactate accumulation in the TME, which enhances m6A modification in tumor-infiltrating myeloid cells (TIMs) through H3K18 lactylation. This activation of the m6A-YTHDF1/JAK1/STAT3 axis is associated with poor prognosis in colon cancer and increases the immunosuppressive capabilities of TIMs (83). Hypoxia in the TME can induce high levels of diacylglycerol kinase gamma in tumor vascular endothelial cells, which in turn can promote tumor angiogenesis and immune evasion in hepatocellular carcinoma through the ZEB2/TGF-β1 axis (84). The Food and Drug Administration’s (FDA) approval of antiangiogenic drugs and, more recently, immunological checkpoint inhibitors has reignited interest in understanding the role of the TME (85). Angiogenesis primarily depends on vascular endothelial growth factor (VEGF). Antiangiogenic drugs are those that inhibit tumor angiogenesis by targeting VEGF, its receptors, and other related molecules (86). The first antiangiogenic targeted medication to target VEGF was bevacizumab, which the FDA approved for application in 2004 (87). Clinical trials have shown that recombinant poliovirus therapy for gliomas, when combined with bevacizumab, had synergistic effects by reducing the local inflammatory response (88). Immune checkpoint inhibitors, including monoclonal antibodies targeting programmed death-1 (PD-1), programmed death-ligand 1 (PD-L1), and cytotoxic T-lymphocyte-associated protein 4 (CTLA-4), counteract the mechanisms tumors use to evade immune surveillance. The first monoclonal antibody targeting CTLA-4, ipilimumab, was approved by the FDA in 2011 for treating melanoma (89). In advanced melanoma patients, combining ipilimumab with a modified oncolytic herpes simplex virus type 1 (HSV-1) demonstrated enhanced anticancer efficacy without additional harm, and these improved response rates persisted at the 5-year follow-up (90).

### 3.2 Tumor-associated immune cells

#### 3.2.1 Cancer-associated fibroblasts

The TME significantly influences the survival, proliferation, migration, and even dormancy of cancer cells (91, 92). It has been established that cancer-associated fibroblasts (CAFs) play a multifaceted role in promoting tumor growth within the TME. CAFs secrete inflammatory ligands, growth factors, and extracellular matrix (ECM) proteins that support tumor growth, contribute to immune exclusion, and foster resistance to treatment (91). As a principal component of the stromal cells, CAFs are recognized for their tumor-promoting properties. However, evidence that CAFs activate the Hedgehog signaling pathway indicates that under certain conditions, CAFs might also exhibit tumor-suppressing functions (93, 94).

Studies have demonstrated that CAFs promote tumor growth through various mechanisms, such as secreting ECM proteins, inducing inflammation and neovascularization, enhancing angiogenesis, increasing the prevalence of tumor-initiating cells, and altering cancer cell signaling (95). Under specific cellular conditions, the multifunctional cytokine TGF-β can both promote and inhibit tumor growth. In pre-menopausal breast cancer, knocking down the TGF-β receptor in cancer-associated fibroblasts (CAFs) has been identified as a predictive factor that can increase the growth, proliferation, and clonogenic survival of breast cancer cells (96). In pancreatic ductal adenocarcinoma, a population of predominantly quiescent CAFs is observed among long-term survivors; those with activated CAFs are highly likely to benefit from therapeutic interventions targeting CAFs (97). In cases of nasopharyngeal carcinoma, CAFs and tumor cells can enhance neoangiogenesis by recruiting endothelial progenitor cells from the bone marrow into the tumor stroma, a process that relies on VEGF and stroma-derived factor-1. Thus, molecules related to angiogenesis may offer viable therapeutic targets in nasopharyngeal carcinom (98). In head and neck cancers (HNCs), fibroblast activation protein (FAP) is expressed by CAFs within the tumor microenvironment. Radiolabeled inhibitors targeting FAP administered to patients with HNCs have demonstrated localized uptake in tumor lesions, indicating activity above background levels (99).

#### 3.2.2 Macrophages

Macrophages are both ubiquitous and specialized across different tissues, playing roles in tissue development, coagulation, inflammation, and every stage of wound healing (100). Macrophages are categorized into two types: immune-suppressive M2 (alternatively activated) and inflammatory M1 (classically activated) (101, 102). Macrophages are essential for immunological homeostasis because they not only play a crucial role in wound healing and tissue repair but also control immune responses through pathogen phagocytosis and antigen presentation. The tumor-promoting actions of immune-suppressive M2 macrophages are enhanced by the TME. A high level of macrophage infiltration in tumors is generally associated with a poor prognosis (103, 104). In the TME, macrophages influence epithelial cell motility, which can be exploited by tumor cells to facilitate their migration and invasion (105).

In prostate cancer, interactions between tumor cells and macrophages are known to facilitate tumor development, although the precise mechanisms remain unclear. It has been found that high-mobility group box 1 activates macrophages, which then produce IL-6. This, in turn, promotes prostate cancer progression, resistance to androgen deprivation therapy (ADT), and gankyrin expression via the STAT3 signaling pathway, creating a self-reinforcing loop. Interestingly, inhibiting the interactions within this loop in a tumor xenograft model has shown to prevent ADT resistance (106). Another study found that patients with metastatic colorectal cancer undergoing chemotherapy that included bevacizumab experienced significantly improved clinical outcomes when they had genetic variations affecting macrophage-related functions. Additionally, alterations in genes related to macrophages may predict the outcome of bevacizumab treatment depending on the KRAS status (107).

#### 3.2.3 Dendritic cells

The innate and adaptive immune systems are bridged by DCs, which are the most potent antigen-presenting cells and are crucial for initiating the adaptive immune response (108). DCs are classified into various subtypes, such as classical DCs, plasmacytoid DCs, and monocyte-derived inflammatory DCs, based on their functional attributes. The cross-priming of tumor-specific T cells by DCs is vital for initiating and sustaining anti-tumor immunity, a process that involves dynamic interactions between different DC subtypes and the tumor (109). The specific type or subtype of the tumor, along with its unique TME, significantly influences the composition and function of DCs. DCs within the tumor are associated with improved patient survival and are capable of inducing T cell responses that enhance protective immunity and decelerate cancer progression (110, 111). Typically, tumors manipulate their environment to ensure their survival, encountering immune-suppressive agents such as VEGF and IL-10 in the TME. These factors hinder the maturation of DCs into immunogenic cells, promoting instead the development of a tolerogenic phenotype in DCs (112, 113).

For some melanoma patients, immunization with mature monocyte-derived DCs)that were loaded with tumor antigens led to tumor regression. Additional refinement of the DC immunization protocol is required to determine which factors contribute to better clinical outcomes and enhanced anti-tumor responses (114). DC vaccines that were transfected with personalized tumor-associated antigen mRNA triggered specific CD4+ and CD8+ T cell responses in patients with advanced lung cancer or glioblastoma multiforme (GBM), and these responses were associated with favorable overall survival without significant autoimmune side effects (115). In a phase I trial, the safety and specificity of immune responses to tumor antigens were evaluated following *in situ* vaccination with autologous DCs transduced with an adenoviral vector expressing the CCL21 gene in patients with advanced non-small cell lung carcinoma (116).

#### 3.2.4 Neutrophils

Neutrophils, constituting up to 70% of circulating leukocytes, serve as the primary line of defense against infections. Typically, they have a short lifespan, remaining in circulation for up to five days. When tissue is infected or damaged, epithelial cells emit chemokines that direct neutrophils to the affected site. Upon arrival, neutrophils deploy extracellular traps (NETs), release inflammatory cytokines, and engulf invading pathogens (117, 118). These NETs, which carry toxins and antimicrobial peptides on a chromatin backbone, serve as an additional mechanism of attack, albeit at the cost of the neutrophil’s own survival (119). The phenotype of neutrophils within the TME varies depending on the type of tumor and the stage of the disease. Initially, during the early stages of tumor development, neutrophils exhibit an inflammatory behavior, but as the tumor progresses, they adopt an immunosuppressive role. Neutrophils manage inflammation by producing ROS and nitrogen species. They also support angiogenesis, tumor invasion, and the remodeling of the ECM by releasing neutrophil elastase and MMPs in the TME. These proteases break down pro-inflammatory cytokines and restructure the TME, fostering tumor growth and metastasis (120, 121).

In the early stages of lung cancer, tumor-associated neutrophils do not suppress the immune system, instead, they enhance T cell responses. This activity leads to a marked increase in costimulatory molecules on the surface of neutrophils, which in turn fosters T cell proliferation in a positive feedback loop (122). Moreover, prior studies have shown that some patients with non-small cell lung cancer exhibit significant anticancer effects after undergoing salvage chemotherapy following PD-1 inhibition. For patients who did not respond to salvage chemotherapy, both the neutrophil-to-lymphocyte ratio (NLR) and the absolute neutrophil count (ANC) increased over the course of treatment with nivolumab. An inverse relationship was observed between the response to the drug and NLR or ANC at four to six weeks (123).

#### 3.2.5 Natural killer cells and natural killer T cells

NK cells are innate lymphoid cells recognized for their cytotoxic capabilities (124). These cells target tumor cells to inhibit the formation of primary tumors through apoptosis induced by death receptors and cytotoxic mechanisms mediated by perforin and granzyme. While NK cells are effective at eliminating circulating tumor cells, they struggle to kill cells within the TME. Tumors deploy various tactics to evade destruction by NK cells, such as surrounding themselves with collagen to trigger inhibitory NK receptors and using platelets as shields to prevent NK cell detection (125, 126). Additionally, many cytokines commonly present in the TME can effectively dampen NK cell effector functions. Natural killer T cells (NKTs) are innate-like T lymphocytes that bind to CD1d and, like conventional T cells, possess a T cell receptor and respond quickly to antigen exposure. These cells are also prevalent within the TME (127). To be more precise, NKTIs may be further classified into subtypes such as Th1-like, Th2-like, Th17-like, regulatory T-like (Treg-like), and T follicular helper (TFH)-like for type I NKTs; whereas type II NKTs can be classified as Th1-like and Th2-like. It has been observed that NKTs can switch roles in the environment, toggling between immune-suppressive and inflammatory roles. Type I NKTs generally exhibit anti-tumor properties, whereas type II NKTs tend to support tumor growth (128, 129).

In ovarian cancer patients, including those from whom cells were isolated from ascites, NK cells demonstrated effective killing of autologous tumor-associated macrophages (TAMs) that expressed low, non-protective levels of HLA class I molecules when appropriately stimulated within the complex TME (130). For achieving pathological complete responses in patients with HER2-positive breast cancer, maintaining functional T cell responses to specific antigens and enhancing NK cell efficacy during neoadjuvant chemotherapy appears crucial (131). Hypoxic TAMs, induced by the tumor, secrete chemokine ligand-21 (CCL21), which attracts and suppresses NKTs. CCL21 further impairs NKT survival and function, inhibiting NKT migration *in vitro* toward tumor-conditioned hypoxic monocytes and preventing their localization to neuroblastoma grafts in mice (132). Patients with asymptomatic myeloma who underwent combination therapy exhibited signs of NK cell activation and an activation-induced reduction in detectable iNKT cells, with the therapy proving effective in driving tumor regression and synergistically activating various innate immune cells (133).

#### 3.2.6 Innate lymphoid cells

Five distinct cell types comprise the innate lymphoid cells (ILCs), including NK cells) (134, 135). Unlike other ILCs, which primarily produce cytokines in reaction to different stimuli, NK cells exhibit the highest cytotoxic activity within the ILC group. These cells are a crucial part of the tumor microenvironment TME (136). ILCs originate from the same lymphoid progenitor as B and T cells but are categorized as innate immune cells due to their lack of B and T cell receptors. They play a role in T cell polarization by presenting antigens and secreting cytokines (137).

Prior to transplantation, the presence of acute leukemia patient ILCs and donor ILCs expressing specific markers was associated with a reduced risk of acute graft-versus-host disease (GVHD) and therapy-induced mucositis. Consequently, the dynamics of ILC recovery and its interaction with treatment-related tissue damage influence the development of GVHD (138). ILCs exhibit a dual role in cancer contexts, showing either pro-tumor or anti-tumor effects depending on the ILC subset and the type of cancer involved. ILC1s, in particular, are an early source of IFN-γ and are generally associated with anti-tumor activity through mechanisms like macrophage activation, Th1 polarization, and enhancement of major histocompatibility complex molecules (139). In cases of metastatic colorectal cancer, the overall frequency of ILCs was markedly increased compared to healthy donors and showed an inverse relationship with Th1 immune responses (140).

## 4 Oncolytic viruses mediate the immune cells and the immune microenvironment

Immunosuppression in the tumor microenvironment, exacerbated by immune checkpoint inhibitors like PD-1/PD-L, diminishes effective neoantigen presentation and hinders anti-cancer T cell responses (141). An engineered oncolytic virus designed to express PD-L1 inhibitors can initiate tumor neoantigen-specific T cell responses. This approach has shown promise in enhancing endogenous T cell reactions against tumor neoantigens in patients with advanced cancers, leading to durable outcomes, including complete responses in various cancer types such as melanoma, and metastatic lung, kidney, and bladder cancers (142, 143).

Oncolytic viruses, capable of inducing anti-tumor immunity both locally and systemically, are either injected directly into the tumor or administered systemically. Once these viruses infect tumor cells, they multiply, often triggering immunogenic cell death, and spread throughout the tumor, stimulating an inflammatory response that can be modulated by macrophages and NK cells of the innate immune system (144). Armed oncolytic viruses also express immunomodulatory transgenes, enhancing immune reactions against the tumor. The immunogenic cell death and inflammation attract DCs to the tumor site, where they initiate a comprehensive immune attack on the tumor by activating T and B cells (145).

Oncolytic viruses can counteract immune evasion strategies utilized by cancer cells. These strategies often involve immuno-inhibitory receptors on tumor cells and in the surrounding microenvironment, which deactivate immune effector cells and promote the secretion of IL-10 and TGF-β. These factors can attract immunosuppressive cells, such as myeloid-derived suppressor cells and tumor-associated macrophages, to the tumor site (146, 147). Oncolytic viruses disrupt this suppressive environment through various mechanisms that alter the cytokine landscape and the composition of immune cells within the TME (148).

Furthermore, several oncolytic viruses possess the capability to express therapeutic genes or alter the function of tumor-associated endothelial cells, enhancing the recruitment of T cells into TMEs that are otherwise immune-deserted or immune-excluded.

## 5 Promisting oncolytic viruses in tumor therapy

### 5.1 Nervous system tumor

Gliomas account for approximately 81% of all central nervous system (CNS) tumors, making them the most common type of malignant brain tumor (149). These tumors are notorious for their aggressive behavior, rapid growth, therapy resistance, and generally poor prognosis. Several non-neurotoxic viruses such as parvovirus, myxoma virus, M1 virus, and Seneca Valley virus are used in treatments as they generally do not require additional modifications for safety (150). The CNS is considered immune-privileged due to the blood-brain barrier (BBB), comprised of astrocytes, pericytes, and vascular endothelial cells forming tight junctions, which typically restricts access of peripheral immune cells to the brain. Overcoming this barrier involves strategies such as direct injection into CNS tumors or using external reservoirs that interface with brain tumor sites. Notably, parvovirus can naturally cross the BBB, facilitating the entry of oncolytic viruses into the bloodstream (151). In GBM clinical studies, parvovirus H-1PV has been utilized. However, there have been relatively few *in vivo* studies on the distribution of oncolytic viruses across the CNS to evaluate viral penetration effectively (152, 153).

### 5.2 Digestive system tumor

Digestive system cancers (DSC), including colorectal cancer (CRC), gastric cancer, hepatocellular carcinoma, pancreatic cancer (PC), and esophageal squamous cell carcinoma, are a leading cause of cancer-related deaths worldwide. Patients with advanced DSC generally have a poor prognosis. Peritoneal metastases, which often occur due to the spread of tumor cells within the peritoneal cavity, commonly triggered by CRC, represent the second most frequent type of CRC metastasis following those in the lung and liver. Approximately 25% of CRC patients develop metastatic disease, and peritoneal metastases are associated with particularly poor outcomes and are a major cause of mortality (154). According to a study, a tumor-lysing vaccinia virus that carries GM-CSF was found to successfully prevent CRC from spreading to the peritoneum by selectively infecting and lysing peritoneal tumor cells, as well as by activating peritoneal DCs and CD8 T cells to restore peritoneal anti-cancer immunity. Various oncolytic viruses, such as vaccine viruses, reoviruses, HSV, adenovirus, oncolytic measles virus, and of virus, are being explored for treating CRC (155). In pancreatic ductal adenocarcinoma, pelareorep, an intravenously administered oncolytic reovirus, has been shown to induce a T-cell-inflamed phenotype in tumors. This treatment has led to T-cell infiltration, increased PD-L1 expression, and active reovirus replication in the tumors of treated patients (59). For gastric cancer, HSV-based OVT has shown promise. The recombinant vaccinia virus used in these treatments has proven safe *in vivo* and demonstrates enhanced replication in tumor cells is an exciting treatment used in OVT for patients with gastric cancer. The recombinant vaccinia virus was safe *in vivo* and had a greater capacity for reproduction in tumor cells (156).

### 5.3 Urogenital system cancer

Prostate cancer is the second most common malignant tumor globally, with an incidence rate of 13.5% and a mortality rate of 6.7%. Current treatments include surgery, hormone therapy, chemotherapy, and radiation therapy (157). However, these treatments often fall short, especially in cases of advanced metastatic prostate cancer. Oncolytic viruses offer a promising alternative due to their high selectivity, efficiency, and low toxicity. These viruses are limited in their ability to replicate in healthy cells but can proliferate within tumor cells, inducing apoptosis and promoting viral growth. The released progeny viruses can then infect adjacent tumor cells, leading to tumor destruction. A phase II study revealed that bladder cancer patients treated with the intravesical oncolytic virus CG0070 experienced a 47% complete response rate at six months (158). Additionally, the bluetongue virus has been shown to infect and selectively lyse human hepatic and prostate cancer cells while significantly increasing the proportion of apoptotic renal cancer cells (159).

### 5.4 Potential clinical applications

There is an increasing body of research on oncolytic viruses that has enhanced our understanding of their role in tumor therapy. Some oncolytic viral therapies, identified as potential prognostic markers and therapeutic targets, have already been approved for clinical use or are advancing into clinical trials for tumor treatment. Currently, single-method treatments for some types of tumors have proven inadequate for achieving optimal results. Oncolytic viruses can inhibit DNA damage repair proteins, thereby minimizing the DNA damage caused by chemoradiation when used in combination with it. Furthermore, chemoradiation can promote tumor cell death, which in turn supports the replication and dissemination of oncolytic viruses. Additionally, combining immunotherapy drugs with oncolytic viruses enhances their synergistic effect, leading to more potent and sustained therapeutic outcomes. Due to their extensive mechanisms of action, oncolytic viruses are vital for intercellular communication and therapeutic applications. It has been observed that insufficient tumor cell tropism and transduction can make the therapeutic virus ineffective when administered systemically in clinical settings. To counter this, modifications to the viral surface have been employed to enhance tumor cell targeting. One such modification is the insertion of an RGD motif into the HI loop of the adenovirus fiber knob domain, which has significantly improved the virus’s infection efficiency and anti-tumor efficacy, especially in CAR-negative tumor models. Additionally, using different virus types, such as Human adenovirus (HAdV)-G52, which binds to polysialic acid on target cells, can offer another layer of targeting specificity. HAdV-G52 might specifically infect cancers, such as brain and lung cancers, that express high levels of polysialic acid, although adjustments are necessary to mitigate any potential neurotropism. Through various modifications, oncolytic viruses can be tailored to enhance their safety, infection capability through tumor cellular receptors, tumor-targeting selectivity, and replication efficiency within tumor cell cytoplasm. These modifications can aid to improve viruses’ immunostimulatory capacity and tissue tropism while maintaining safety and antitumor efficiency.

## 6 Conclusion

There are several challenges with the oncolytic viral therapy approach. Research on oncolytic viruses is still in its infancy. Many oncolytic viruses are currently being explored both theoretically and experimentally, and their potential therapeutic value remains uncertain. Additionally, there are no specific diagnostic criteria to identify patients who might benefit from oncolytic viral therapies.

In conclusion, the function of oncolytic viruses in tumor therapy and the tumor microenvironment is gradually becoming apparent. As either naturally occurring or genetically engineered agents, oncolytic viruses achieve their therapeutic effects by influencing immune cells, tumor cells, and the tumor microenvironment. This review has discussed the history and characteristics of the tumor microenvironment, immune cells, and oncolytic viruses, as well as the potential therapeutic efficacy of oncolytic viruses. These viruses activate various pathways to induce tumor cell death. A significant challenge in cancer treatment is that many oncolytic virus therapies are still in the preclinical trial stage. While a few oncolytic viruses have been approved for clinical use, transitioning these therapies into clinical applications remains a substantial hurdle. Consequently, understanding how oncolytic viruses modify the tumor microenvironment in different tumors will lead to the enhancement and development of oncolytic virus treatments for cancer. Moving forward, continued research into oncolytic viruses is essential.

## Glossary

OVT: Oncolytic virus therapy
TME: Tumor microenvironment
ROS: Reactive oxygen species
ECM: Extracellular matrix
CAF: Cancer-associated fibroblast
DC: Dendritic cell
NK: Natural killer cell
NKT: Natural killer T cell
MMP: Matrix metalloproteinase
m6A: RNA N6-methyladenosine
TIM: Tumor-infiltrating myeloid cells
FDA: Food and Drug Administration
VEGF: Vascular endothelial growth factor
PD-1: Programmed death-1
PD-L1: Programmed death-ligand 1
CTLA-4: Cytotoxic T-lymphocyte-associated protein 4
HSV-1: Herpes simplex virus type 1
FAP: Fibroblast activation protein
HNC: Head and neck cancer
ADT: Androgen deprivation therapy
CCL21: Chemokine ligand-21
TAM: Tumor-associated macrophages
NLR: Neutrophil-to-lymphocyte ratio
ANC: Absolute neutrophil count
ILC: Innate lymphoid cells
GVHD: Graft-versus-host disease
CNS: Central nervous system
BBB: Blood-brain barrier
DSC: Digestive system tumor
PC: Pancreatic cancer
CRC: Colorectal cancer
HAdV: Human adenovirus
